# Sedentary behavior during leisure time, physical activity and dietary habits as risk factors of overweight among school children aged 14–15 years: case control study

**DOI:** 10.1186/s13104-018-3292-y

**Published:** 2018-03-20

**Authors:** Indrani Godakanda, Chrishantha Abeysena, Ayesha Lokubalasooriya

**Affiliations:** 1 Ministry of Health, Colombo, Sri Lanka; 2 Department of Public Health, Faculty of Medicine, University of Kelaniya, Kelaniya, Sri Lanka; 3 Adolescents Health Unit, Family Health Bureau, Colombo, Sri Lanka

**Keywords:** Diet, Leisure time, Overweight, Physical activity, Sedentary

## Abstract

**Objective:**

To determine the risk of sedentary behavior during leisure time, physical activity and dietary habits on overweight among school children aged 14–15 years in Kalutara District, Sri Lanka.

**Results:**

School based case–control study was conducted during September to November 2013 including 176 overweight children as cases and 704 children with normal weight as controls. Cases were defined as body mass index for age and sex of ≥ +1SD and controls as those in the range of −2SD to +1SD. Validated instruments were used for data collection. Multiple logistic regression was applied and results were expressed with adjusted odds ratios (OR) and 95% confidence intervals (CI). Risk factors for overweight were insufficient physical activity (OR 1.6, 95% CI 1.1–2.4), watching video/DVD ≥ 2 h (OR 3.1, 95% CI 1.8–5.3), watching television ≥ 2 h (OR 2.6, 95% CI 1.7–3.8) and doing homework ≥ 2 h, (OR 1.8, 95% CI 1.2–2.7). Consuming meat (OR 1.9, 95% CI 1.2–3.1), fish or other sea foods (OR 1.6, 95% CI 1.1–2.8), fast food/fried rice/oily foods (OR 1.9, 95% CI 1.2–2.9), carbonated drinks or sugary drinks (OR 1.9, 95% CI 1.2–2.8), sweets, cookies or ice cream (OR 1.8, 95% CI 1.2–2.9) were dietary risk factors for overweight. Consuming legumes and seeds (OR 0.50, 95% CI 0.3–0.7), vegetables and fruits (OR 0.6, 95% CI 0.4–0.9) were protective factors for overweight.

## Introduction

A few years back overweight and obesity were considered as a problem found mainly in high income countries. However, it is now dramatically on the rise in low- and middle-income countries as well [1]. The nutrition transition in developing countries shifted people from their traditional diet based on cereals, legumes, vegetable and fruits to an energy dense diet, rich in saturated fatty acids, salt and sugar. In addition to changing, the dietary behaviours declining physical activities with increased time spent in sedentary activities are major factors underlying childhood obesity [2]. A review [3] reported that the risk of overweight/obese youth becoming overweight/obese adults is increased by 75%.

Sedentary behaviour is not simply a lack of physical activity, instead of individual behaviours where sitting or lying is the dominant mode of posture and where energy expenditure is very low [4]. Assessment of sedentary behavior includes the time spent in a comprehensive range of sedentary activities [5]. The literature emphasized the distinction between sedentary behaviour and the absence of moderate and vigorous physical activities (MVPA) [6, 7]. A systematic review revealed that sedentary behaviors, as measured by total screen-viewing time, lower fruit and vegetable intake and higher consumption of energy-dense snacks, and fast foods were associated with overweight [8]. One study revealed that sedentary behavior was inversely associated with risk of overweight/obesity [9]. However, another study found that total sedentary time spent was not associated with overweight/obese independent of MVPA [10]. Further, case control or a cohort studies are more suitable for assessment of risk factors of a disease rather than descriptive studies.

Adolescence, considered as a decisive period in life is the period of transition from childhood to adulthood. It is defined as the age group from 10 to 19 years [11]. Adolescent population consists of 18.8% of the total population in Sri Lanka [12]. At present Sri Lankan adolescents too are being influenced by a range of individual and environmental transitions. In relation to the Sri Lankan context extremely limited number of studies published in related to sedentary activities. The purpose of this study was to determine the risk of sedentary behavior during leisure time, physical activity and dietary habits on overweight among school children aged 14–15 years in Kalutara District, Sri Lanka.

## Main text

### Methods

A case control study was carried out in Grade 9 and 10 in the schools of Kalutara District in Sri Lanka during September to November 2013. A case was defined as a child who had a body mass index (BMI) for age and sex of ≥ +1SD (overweight) according to the growth chart of World Health Organisation [13]. A control was defined as a child who had a BMI for age and sex in the range of −2SD to < +1SD (normal weight) [13]. Children who had past history of overweight management such as diet control, or any other treatment or any health education related to overweight were excluded.

Multi-stage cluster sampling method with probability proportionate to the size was applied considering the large geographical area and variability of schools by sector and functional type. A class was considered as a cluster. For the sample size calculation the proportion of sedentary behavior among controls was considered as 16% [14], with a design effect of 1.3, odds ratio of 2, power of 80% and ${\text{Z}}_{{ - {{1\upalpha} \mathord{\left/ {\vphantom {{1\upalpha} 2}} \right. \kern-0pt} 2}}}$ of 0.05. Case to control ratio was taken as 1:4. Therefore, the required sample size was 169 cases. Assuming non responses rate of 5%, the final sample size required for recruitment was 176 for the cases and 704 for the controls.

Selection of cases and controls were carried out independent of knowledge on their exposure status. The anthropometric measurements were made using standardized equipment and standard procedures. The same instruments were used throughout the study and all equipment were calibrated prior to the commencement of taking measurements at each session. The circumstances under which data were collected from cases and controls were comparable.

Validated Adolescents Sedentary Activity Questionnaire-Sinhala [15] and Physical Activity Questionnaire-Sinhala [16] were used for assessing sedentary and physical activities respectively. Validated 3-day dietary record [16] was used to assess dietary constituents on each day. Parental questionnaire was used to collect data of the parents. All participants were asked to recall their daily activities (before and after school) and the time spent on each of the different activities during the 7 days. Data collection was done during term time in a week devoid of any extracurricular activities or examinations to ensure minimum information bias.

Pattern of sedentary activities was assessed in minutes spent on each activity on each day of the week. Sedentary behaviour was assessed by summing up the total sedentary hours spent per day and which was dichotomized [15] as ‘more sedentary’ and ‘less sedentary’ (total sedentary activities ≥ 4 and < 4 h per day respectively). In addition, each sedentary activity was categorized into two ≥ 2 and < 2 h. A participant in MVPA for at least 60 min per day for ≥ 5 day per week were categorized as ‘sufficiently active’ and otherwise as ‘insufficiently active’ [16]. Each food item was categorized under one of the 25 food domains and a raw score was assigned. Each raw score was grouped under 11 major food groups based on Food and Agriculture Organization (FAO) guidelines [17] and these include as legumes and seeds; vegetables; dark green leaves; fruits; meat; fish or other sea foods; eggs; milk; fast foods, fried rice, oily foods; carbonated drinks, sugary drinks, ice packets; sweets, biscuits or ice cream. Consumption of each food group was categorized as ‘taken’ or ‘not taken’.

Bivariate analysis was carried out to assess the association of each probable risk factor and being overweight. Variables that showed a probability value of < 0.20 in the bivariate analysis were considered as eligible variables for the regression model. Multiple logistic regression was performed to identify the independent risk of each variable with overweight. Purposeful selection method was performed. Results were expressed as odds ratios (OR) and the respective 95% confidence intervals (CI).

### Results

Majority (67.7%, n = 596) of the participants were 14 years old. Among the cases 52.8% (n = 93) were males and among the controls the figure was 54% (n = 380). Majority of participants (63%. 110 cases versus 70.6%, 497 controls) were from rural schools. Being unemployed (p = 0.01) and higher educational level of the mother (p = 0.02) and higher family income (p = 0.02) were associated with increased risk of overweight.

Sedentary activities were associated with higher risk of overweight except participating ≥ 2 tuition/extra classes per day, performing music for ≥ 2 h per day and not playing for sports clubs during the last 12 months (Table 1).

**Table 1:** Unadjusted odds ratios for sedentary and physical activities and overweight among adolescents

Sedentary and physical activities	Cases (n = 176)	Controls (n = 704)	OR (95% CI)	p value
	Frequency %	Frequency %		
Total sedentary time ≥ 4 h/day (more sedentary)	170 (96.6)	637 (90.5)	2.9 (1.3–6.9)	0.009
Radio listening time ≥ 2 h/day	163 (92.6)	636 (90.3)	1.4 (0.7–0.9)	0.03
TV watching time ≥ 2 h/day	93 (52.8)	248 (35.2)	2.06 (1.5–2.9)	< 0.01
Video/DVD time ≥ 2 h/day	33 (18.8)	78 (11.1)	1.8 (1.2–2.9)	0.006
Computer working time ≥ 2 h/day	23 (13.1)	41 (5.8)	2.4 (1.4–4.2)	0.001
Reading for fun time ≥ 2 h/day	18 (10.2)	11 (1.6)	7.1 (3.3–15.5)	< 0.01
Home work time ≥ 2 h/day	58 (33.0)	167 (23.7)	1.6 (1.1–2.3)	0.012
Tuitions/extra class time ≥ 2 h/day	21 (11.9)	76 (10.8)	1.2 (0.67–1.9)	0.60
Phone usage time ≥ 2 h/day	8 (4.5)	7 (1.0)	4.7 (1.6–13.2)	0.001
Music doing time ≥ 2 h/day	2 (1.1)	7 (1.0)	1.1 (0.23–5.6)	0.80
Insufficiently physical active	117 (66.5)	399 (56.7)	1.5 (1.1–2.1)	0.018
Going to school by foot cycle ≤ 2 days/week	103 (58.5)	339 (48.2)	1.5 (1.1–2.1)	0.014
No of sports clubs played for last 12 months—none	69 (39.2)	247 (35.1)	1.2 (0.8–1.7)	0.30
Participated in sport clubs, aerobics, dancing classes for ≤ 2 days/week last 12 months	154 (87.5)	568 (80.7)	1.7 (1.1–2.7)	0.03
Participated in school morning physical exercise programme ≤ 2 days/last week	171 (97.2)	654 (92.2)	2.6 (1.1–6.7)	0.014

OR, odds ratio; CI, confidence interval

Of the 176 cases, 151 responded satisfactorily to the 3 day dietary record. Out of the 704 controls 617 responded the 3-day dietary record. Of them 604 controls were randomly selected to keep the case–control ratio of 1:4. Consumption of all food items except consumption of dark green vegetables, eggs and milk, were associated with a higher risk of being overweight (Table 2).

**Table 2:** Unadjusted odds ratios for food consumption and overweight among adolescence

Consumption of food items	Cases (151)	Controls (604)	OR (95% CI)	p value
	Frequency %	Frequency %		
Legumes and seeds	115 (76.2)	511 (84.6)	0.58 (0.4–0.8)	0.01
Dark green leaves	94 (62.3)	393 (65.1)	0.8 (0.6–1.3)	0.50
Vegetables	161 (66.9)	459 (76)	0.6 (0.43–0.9)	0.02
Fruits	55 (36.4)	276 (45.7)	0.68 (0.5–0.9)	0.04
Meat	46 (30.5)	117 (19.3)	1.8 (1.2–2.7)	0.003
Fish, other sea foods	112 (74.2)	389 (64.4)	1.5 (1.1–2.4)	0.023
Eggs	41 (27.2)	157 (26)	1.1 (0.7–1.6)	0.70
Milk	122 (80.8)	501 (82.9)	0.9 (0.5–1.36)	0.50
Fast foods, fried rice, oily foods	112 (74.2)	356 (60)	1.9 (1.3–2.8)	0.001
Carbonated drinks, sugary drinks	57 (37.7)	150 (24.8)	1.8 (1.3–2.7)	0.001
Sweets, cookies, ice cream	107 (70.9)	364 (60.3)	1.6 (1.1–2.4)	0.017

OR, odds ratio; CI, confidence interval

Results of multiple logistic regression analysis is shown in Table 3. Risk factors for overweight were watching video/DVD for ≥ 2 h (OR 3.1, 95% CI 1.8–5.3), watching television for ≥ 2 h (OR 2.6, 95% CI 1.7–3.8), doing homework for ≥ 2 h (OR 1.8, 95% CI 1.2–2.7) and insufficient physical activity (OR 1.6, 95% CI 1.1–2.4). Consumption of meat (OR 1.9, 95% CI 1.2–3.1), fish or other sea food (OR 1.6, 95% CI 1.1–2.8), fast food, fried rice and oily foods (OR 1.9, 95% CI 1.2–2.9), carbonated drinks, sugary drinks and ice packets (OR 1.9, 95% CI 1.2–2.8) demonstrated higher risk for overweight in comparison to controls. Consumption of legumes and seeds (OR 0.50, 95% CI 0.3–0.7), vegetables and fruits (OR 0.60, 95% CI 0.4–0.9) demonstrated a protective effect for overweight.

**Table 3:** Adjusted odds ratios for risk factors and overweight among adolescence

Risk factors	Β	SE (β)	OR	95% CI for OR		p value
				Lower	Upper	
Television watching time ≥ 2 h/day	0.94	0.2	2.6	1.7	3.8	< 0.001
Video/DVD watching ≥ 2 h/day	1.12	0.28	3.1	1.8	5.3	< 0.001
Homework ≥ 2 h/day	0.59	0.22	1.8	1.2	2.7	< 0.001
In sufficiently physical activity	0.45	0.21	1.6	1.1	2.4	0.03
Legumes and seeds consume	− 0.75	0.25	0.5	0.3	0.7	0.003
Vegetables consume	− 0.47	0.22	0.6	0.4	0.9	0.03
Fruits consume	− 0.45	0.21	0.6	0.4	0.9	0.03
Meat consume	0.66	0.24	1.9	1.2	3.1	0.007
Fish, other sea foods consume	0.57	0.24	1.6	1.1	2.8	0.01
Fast food, fried rice, oily foods consume	0.63	0.23	1.9	1.2	2.9	0.006
Carbonated drinks, sugary drinks, ice packets consume	0.62	0.22	1.9	1.2	2.8	0.005
Sweets, cookies, ice cream consume	0.59	0.22	1.8	1.2	2.7	0.007
Mothers education O/L and above	0.64	0.22	1.9	1.3	2.8	0.003

β-Regression coefficient; SE (β) standard error of β; CI, confidence interval; OR, adjusted odds ratio. The Hosmer and Lameshow test χ^2^ = 2.4 (p value = 0.97)

### Discussion

We found that watching television for ≥ 2 h per day and video/DVD for ≥ 2 h per day were risk factors for overweight. Several studies have revealed similar findings despite using different study designs, settings and cutoff values of exposure status [8–20]. Those who spent ≥ 2 h of homework demonstrated 1.8 times risk for overweight in our study. Though evidence related to risk of homework time for overweight is unavailable, students are also expected to engage in sedentary behaviour in the form of homework/educational activities.

However, we could not find an association between spending ≥ 4 h of total sedentary time (more sedentary) and overweight. We found that unadjusted odds ratio was statistically significant. Our findings are consistent with two other studies [10, 21], revealed that sedentary behavior was not a risk factor for overweight/obesity, independent of the VMPA. In contrast, a case control study revealed that ≥ 4 h of total sedentary time was a risk factor for overweight/obesity [9]. Those who were physically inactive were 1.6 time at a higher risk of overweight than those who were sufficiently active. The findings are comparable to the results of number of studies [9, 10, 21, 22].

Present study too revealed that consuming pulses and seeds provides a protective effect against overweight. It was same for consuming fruits and vegetables. Our findings are consistent with several other studies conducted among adolescents as well as among adults in different geographical areas [20, 23, 24]. Fruits and vegetables are of low energy density and therefore a larger volume of food has to be consumed to obtain a certain level of calories.

In the present study meat consumption showed a positive association with overweight with an odds ratio of 1.9. This finding has been confirmed by another study [19]. Consumption of fish or other sea food varieties demonstrated a positive association with overweight in the present study. Suematsu et al. [25] showed that the incidence of out-of-hospital cardiac arrests was positively associated with the consumption of tuna, salmon, saury, and cuttlefish. Though fish intake is recommended as a good dietary behavior, consumption of fried fish, pawns, crabs and cuttlefish can be associated with overweight.

We found that those who consume fast food, fried rice and oily foods were 1.9 times of a higher risk of becoming overweight. Several studies from different geographical locations reported that consumption of fast foods have a higher risk of weight gain, overweight or obesity [19, 24, 25]. These foods typically contain potentially adverse dietary factors including saturated and trans-fat, high glycemic index, high energy density, and, increasingly large portion size. Additionally, these foods tend to be low in fiber, micronutrients, and antioxidants [26].

We found that those who consume carbonated drinks sugary drinks and ice packets were also at higher risk of overweight. Two other studies also reported that sugar-sweetened beverage were associated with a higher risk of overweight or obesity [19, 21]. In addition, those who consume of sweets, biscuits and ice cream demonstrated too are a higher risk of (1.8 times) overweight. However, contradictory to our findings, two studies reported that higher sweet intake was negatively associated with overweight/obesity [19, 27]. This may be due to the fact that overweight adolescents are more likely to under-report unhealthy food intake or they have changed their behavior and consume less sweets because of their concern of being overweight [28].

The study was carried out in all three educational zones in the District of Kalutara enabling the generalization of the study findings to all the schools in the district.

## Limitations

One of the limitations was difficulty in establishing temporal relationship between the exposures and overweight because of the cross-sectional nature of a case control study. Even though we used validated and reliable self-administrated questionnaires, there would be the tendency to under or over report exposures. In addition, the possibility of recall bias could not be totally excluded. However, objective measures were not feasible for assessing exposures on behaviours. A school based high quality interventional study which assess effectiveness of educational package would be recommended.

## Abbreviations

BMI: body mass index
CI: confidence interval
FAO: Food and Agriculture Organization of the United Nation
MVPA: moderate to vigorous intensity physical activity
OR: odds ratio
SD: standard deviation
WHO: World Health Organization
